# Interferon signaling in *Peromyscus leucopus* confers a potent and specific restriction to vector-borne flaviviruses

**DOI:** 10.1371/journal.pone.0179781

**Published:** 2017-06-26

**Authors:** Adaeze O. Izuogu, Kristin L. McNally, Stephen E. Harris, Brian H. Youseff, John B. Presloid, Christopher Burlak, Jason Munshi-South, Sonja M. Best, R. Travis Taylor

**Affiliations:** 1 Department of Medical Microbiology and Immunology, University of Toledo College of Medicine and Life Sciences, Toledo, Ohio, United States of America; 2 Innate Immunity and Pathogenesis Unit, Laboratory of Virology, Rocky Mountain Laboratories, DIR, NIAID, NIH, Hamilton, Montana, United States of America; 3 The Graduate Center, City University of New York, New York, New York, United States of America; 4 Department of Surgery, University of Minnesota, Minneapolis, Minnesota, United States of America; 5 Louis Calder Center-Biological Field Station, Fordham University, Armonk, New York, United States of America; University of Texas Medical Branch at Galveston, UNITED STATES

## Abstract

Tick-borne flaviviruses (TBFVs), including Powassan virus and tick-borne encephalitis virus cause encephalitis or hemorrhagic fevers in humans with case-fatality rates ranging from 1–30%. Despite severe disease in humans, TBFV infection of natural rodent hosts has little noticeable effect. Currently, the basis for resistance to disease is not known. We hypothesize that the coevolution of flaviviruses with their respective hosts has shaped the evolution of potent antiviral factors that suppress virus replication and protect the host from lethal infection. In the current study, we compared virus infection between reservoir host cells and related susceptible species. Infection of primary fibroblasts from the white-footed mouse (*Peromyscus leucopus*, a representative host) with a panel of vector-borne flaviviruses showed up to a 10,000-fold reduction in virus titer compared to control *Mus musculus* cells. Replication of vesicular stomatitis virus was equivalent in *P*. *leucopus* and *M*. *musculus* cells suggesting that restriction was flavivirus-specific. Step-wise comparison of the virus infection cycle revealed a significant block to viral RNA replication, but not virus entry, in *P*. *leucopus* cells. To understand the role of the type I interferon (IFN) response in virus restriction, we knocked down signal transducer and activator of transcription 1 (STAT1) or the type I IFN receptor (IFNAR1) by RNA interference. Loss of IFNAR1 or STAT1 significantly relieved the block in virus replication in *P*. *leucopus* cells. The major IFN antagonist encoded by TBFV, nonstructural protein 5, was functional in *P*. *leucopus* cells, thus ruling out ineffective viral antagonism of the host IFN response. Collectively, this work demonstrates that the IFN response of *P*. *leucopus* imparts a strong and virus-specific barrier to flavivirus replication. Future identification of the IFN-stimulated genes responsible for virus restriction specifically in *P*. *leucopus* will yield mechanistic insight into efficient control of virus replication and may inform the development of antiviral therapeutics.

## Introduction

Flaviviruses such as dengue virus (DENV), West Nile virus (WNV), Zika virus (ZIKV), and tick-borne flaviviruses (TBFVs) are globally important human pathogens that cause significant morbidity and mortality. These viruses are classified by the mode of transmission (mosquito-borne, tick-borne, or no known vector), as well as disease outcomes, causing encephalitis or hemorrhagic fever [1]. No specific treatment for clinically recognized cases exists for any flavivirus and few vaccines are available [2,3]. As with many zoonotic pathogens, flaviviruses are largely maintained in nature without causing disease in their specific reservoir host [4] yet the same pathogens can have devastating effects on human hosts during infection. Understanding the infectious dichotomy between humans and natural hosts may ultimately reveal genetic determinants involved in protection from severe disease and inform new therapeutic strategies.

TBFVs are regarded as emerging pathogens [5] defined by a re-emergence in historic regions and case reports in areas where they were not previously associated with disease [6–9]. Although the exact reason for the increased and continuous virus spread is not understood [2], hypotheses to explain the emerging state of TBFVs include changes to urban migration, socio-economic dynamics, vector and host ecology, and modifications to the virus genome that increase virulence and pathogenesis [2,10–12]. Powassan virus (POWV lineage I) and deer tick virus (POWV lineage II) are the only recognized TBFVs in North America [13,14]. Human disease due to POWV is similar to other tick-borne encephalitic flaviviruses and can result in severe neurological complications and fatality in 60% of patients that present encephalitis [3,14]. There has been a steady increase in the incidence of POWV infection of humans that demands a closer examination of the determinants of virus spread and pathogenesis [13,14]. In nature, POWV (and other TBFVs) is maintained in ixodid ticks that must feed on mammals in order to reproduce and molt into their next life stage [15]. Tick larvae and nymphs feed on small mammals including rodents, whereas adult ticks feed on larger animals, including deer. Although it is arguable whether ticks are the true reservoirs of TBFVs rather than a mammal, rodents are essential for virus transmission between feeding ticks and maintenance of the virus in nature [15,16]. Historically, reservoir host species have been defined by the identification of virus or virus-specific antibodies in wild-caught rodents with no apparent disease symptoms. *Peromyscus leucopus*, the white-footed mouse, is the most abundant rodent species in North America. Importantly, *P*. *leucopus* is naturally infected with POWV [13,17–19] as well as with a variety of bacterial pathogens (*Borrelia*, *Anaplasma*, *and Rickettsia* [20–23]) and other viruses (hantaviruses [24–26]) without succumbing to infection [17]. Thus, *P*. *leucopus* may function as a reservoir host for POWV.

Laboratory mice (*Mus musculus*) are useful in the modeling of flavivirus encephalitis, including POWV, as they are susceptible to infection and recapitulate many aspects of human disease, including the identity of infected cells and organs, induction of inflammatory responses, and damage to the central nervous system [14]. *Peromyscus* mice differ significantly from both the *Mus* and *Rattus* genera having diverged an estimated 22 million years ago [27,28]. Laboratory studies reveal that the outcome of infection with POWV is markedly different; wild-caught *P*. *leucopus* mice experimentally infected with POWV show no signs of encephalitis or death [18]. A direct *in vivo* comparison of POWV replication between *P*. *leucopus* and susceptible *Mus* counterparts was performed recently. The study systematically demonstrated that intraperitoneal and intracranial infection of *P*. *leucopus* mice leads to low viral load, limited viral spread in the brain and overall survival of infection while control C57BL/6 and BALB/c mice showed higher viral load, increased viral spread, demonstrated neurological symptoms and ultimately died from infection [29]. These studies further implicate *P*. *leucopus* as a natural reservoir host of TBFV infection. The dichotomy of infection outcomes between *M*. *musculus* and *P*. *leucopus* infected with POWV provides an ideal opportunity to examine the mechanism of restriction of TBFVs in *P*. *leucopus* hosts. Detailed molecular studies to delineate the genetic determinants of reservoir host protection do not exist because of the absence of a well-defined cell culture model of TBFV restriction.

Based on disease resistance in *P*. *leucopus*, we hypothesized that the reservoir species has developed potent antiviral factors that restrict virus infection to prevent disease progression. We tested this hypothesis at the cellular level by comparing replication of multiple vector-borne flaviviruses in primary fibroblasts derived from either *P*. *leucopus* or *M*. *musculus* C57BL/6 mice to begin to understand the genetic basis of flavivirus restriction. Our data revealed that *P*. *leucopus* mouse cells potently restrict both TBFV and mosquito-borne flavivirus (MBFV) replication. Restriction is dependent upon signaling by type I interferon (IFN), as virus replication is increased when the host response was blunted. The TBFV-*Peromyscus* model established in this study provides a framework for further investigation into virus-reservoir model pairs.

## Results

### Construction of an *in vitro* model to study TBFV infection in a natural host

The TBFV transmission cycle involves wild rodents, and evidence in the literature suggests that the virus is maintained in the natural host without causing overt disease [18,30–32]. The overall goal of our study was to develop a model to study TBFV interactions with a reservoir host at a cellular and molecular level. We obtained primary adult skin fibroblasts derived from closed colony outbred *P*. *leucopus* mice at the *Peromyscus* Genetic Stock Center (PGSC). In addition, we expanded primary embryonic fibroblasts (*Peromyscus* embryonic fibroblasts, PEFs) from 13 day *P*. *leucopus* embryos also obtained from the PGSC. Morphologically, both adult fibroblasts and PEFs were indistinguishable from human or rodent fibroblast cultures. We chose mouse embryonic fibroblast cells (MEFs) from the C57BL/6 strain of *M*. *musculus* for comparison as an extensively used cell type in flavivirus research.

### Vector borne flaviviruses are specifically restricted in *P*. *leucopus* cells *in vitro*

**Fig 1:**
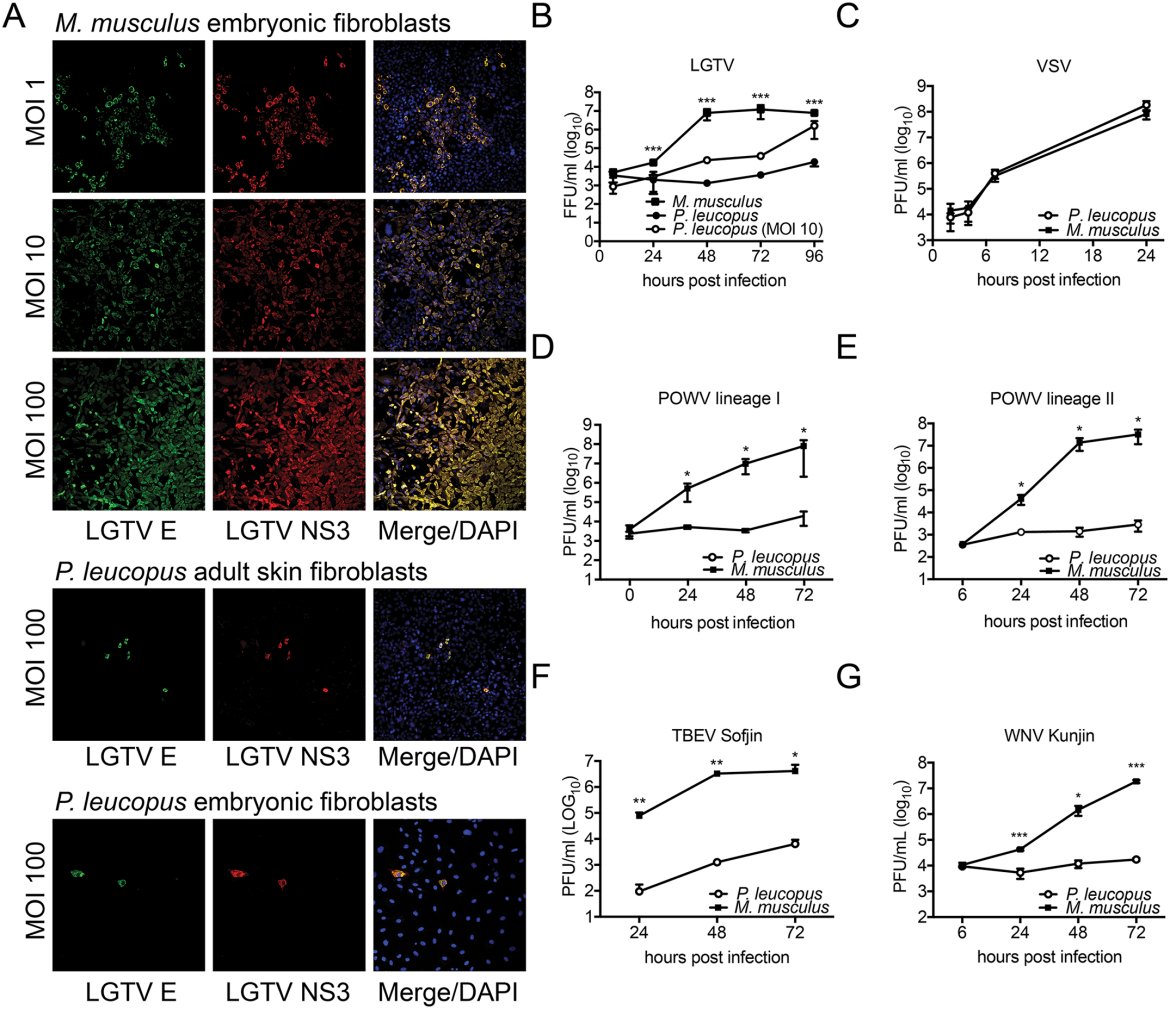
Vector-borne flaviviruses are specifically restricted in *P*. *leucopus* cells *in vitro*. (A) Adult skin primary fibroblasts and embryonic fibroblasts from *P*. *leucopus* and *M*. *musculus* (respectively) were infected with LGTV and assessed by immunofluorescence and probed for E and NS3 proteins. Cell nuclei are stained in blue (DAPI). 20X magnification. *P*. *leucopus* and *M*. *musculus* fibroblasts were infected with: (B) LGTV (MOI 1 and 10), (C) VSV, (D) POWV-I, (E) POWV-II, (F) TBEV Sofjin, and (G) WNV Kunjin. Supernatants were collected at the indicated time points and quantified by immunofocus or plaque assay. Immunofocus assay data are presented as virus focus-forming units per ml (FFU/ml), while plaque assay data are presented as plaque forming units per ml (PFU/ml). Mean ± SD, data are from three independent experiments performed in triplicate. Asterisks indicate: * = p < 0.05, ** = p <0.01, *** = p <0.0001.

To determine whether TBFVs can productively infect cells from *P*. *leucopus*, we initially used Langat virus (LGTV), a prototypic TBFV that is routinely used as a biosafety level (BSL)-2 model for TBFVs [32,33]. By confocal microscopy, *M*. *musculus* fibroblasts were clearly infected with LGTV, as assessed by virus-specific envelope (E) and nonstructural protein 3 (NS3) protein staining (Fig 1A). By 72 hours post infection (hpi), the entire culture appeared to be infected at the high multiplicity of infection (MOI). In contrast, reduced numbers of infected adult or embryonic *P*. *leucopus* cells were observed, though viral structural and nonstructural protein staining was clearly evident in the infected cells, which is suggestive of virus replication.

Titration of infectious LGTV produced from *P*. *leucopus and M*. *musculus* fibroblasts supported the findings by microscopy. At an MOI of 1, up to 10,000-fold less virus was produced from *P*. *leucopus* cells in comparison to cultures from *M*. *musculus* (Fig 1B). This difference was reduced when increased LGTV MOI was used, although significantly more virus was produced from *M*. *musculus* cells even with a 10-fold difference in initial inoculum. The increase in virus production with increasing MOI suggests that *P*. *leucopus* fibroblasts support active, albeit reduced, virus replication. A similar restriction in LGTV replication was observed in PEFs (data not shown). In contrast, fibroblasts from both mouse species supported equal replication of vesicular stomatitis virus (VSV, Fig 1C). VSV is a negative-sense RNA rhabdovirus that causes encephalitis similar to TBFVs in mice [34,35]. The lack of VSV restriction indicates that the *P*. *leucopus* cells are not resistant to all virus infections and suggests that restricted permissiveness is specific to certain viruses.

**Fig 2:**
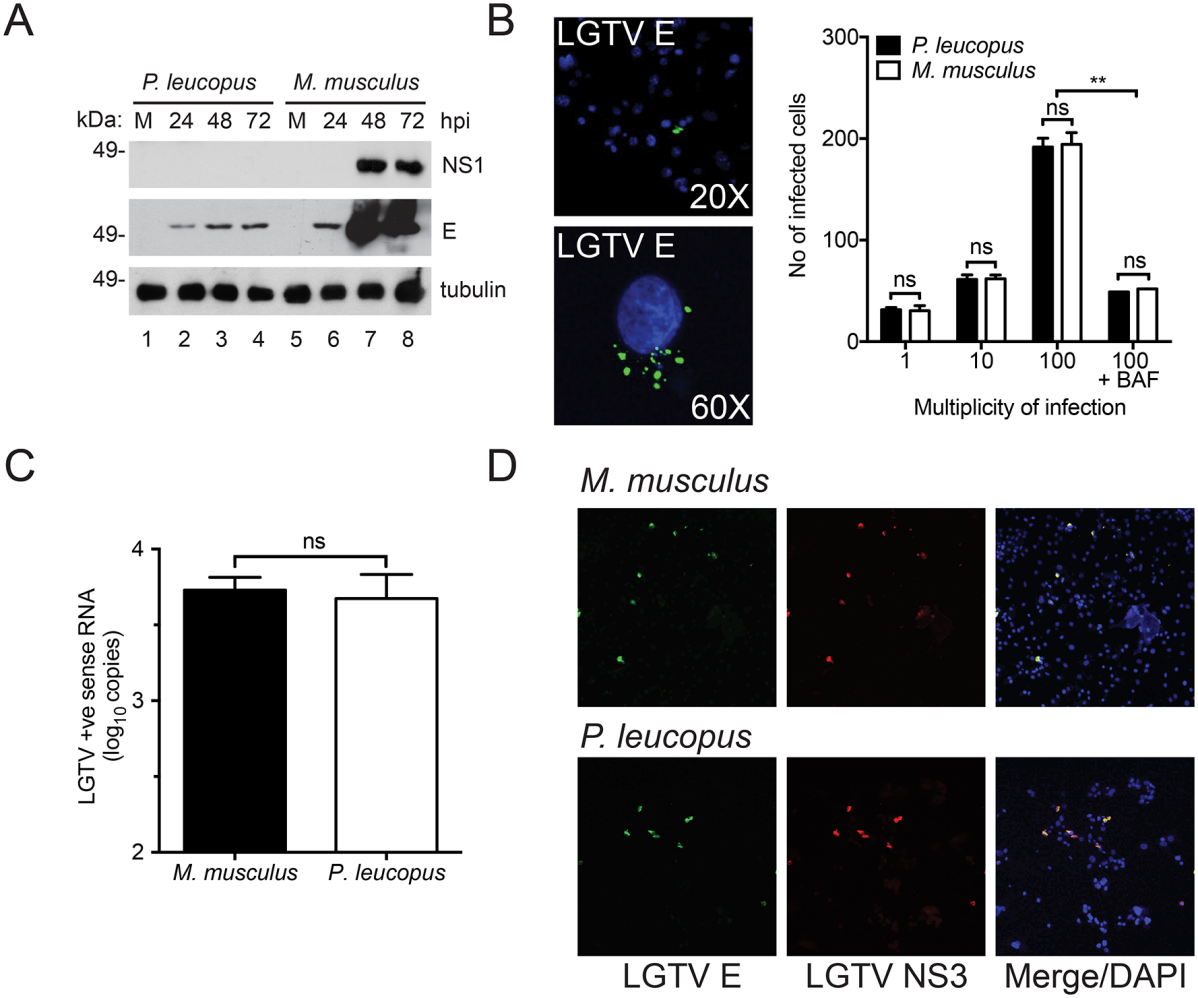
TBFV entry and protein expression are not inhibited in *P*. *leucopus* cells. (A) Immunoblot of LGTV NS1 and E proteins in *P*. *leucopus* and *M*. *musculus* fibroblasts at the indicated time points post infection. (B) Representative confocal 20X (top) and 60X (bottom) images showing *P*. *leucopus* cells infected with LGTV after 1 hpi at 37°C. The viral E protein is stained in green and nuclei are stained in blue (DAPI). Quantified data are shown as the total number of infected cells at MOI 1, 10, and 100 respectively. Cells treated with bafilomycin A1 (BAF) at 15 min post-infection are shown as a negative control. (C) Viral entry assay showing abundance of LGTV positive (+ve) strand RNA in *P*. *leucopus* and *M*. *musculus* fibroblasts. The cells were infected at MOI 10 and incubated at 4°C for 1 h before a temperature shift to 37°C for another h. Total RNA was harvested following an acid wash and the resultant cDNA was used as template for RT-qPCR. (D) Confocal image showing *P*. *leucopus and* C57BL/6 expressing LGTV proteins. Cells were transfected with viral RNA for 5 days after which the cells were fixed and immunostained for LGTV E (green) and NS3 (red). Cell nuclei were stained with DAPI (blue) and visualized by confocal microscopy (20X magnification).

To extend these findings, we examined the replication of more virulent TBFVs in *P*. *leucopus* cells. The BSL-3 POWV lineage I (Fig 1D) and POWV lineage II (Fig 1E), as well as the BSL-4 TBEV (Fig 1F) were all restricted to similar levels observed with LGTV. Finally, we examined replication of a MBFV, WNV Kunjin, and observed similar restriction (Fig 1G) suggesting that restriction is specific to the flavivirus family. Western blotting for NS1 and E proteins following LGTV infection revealed higher accumulation of LGTV proteins over the course of infection in *M*. *musculus* compared to *P*. *leucopus* cells. (Fig 2A) demonstrating that restriction in virus production corresponds to reduced accumulation of viral proteins. Taken together, these data show that both TBFVs and a MBFV, WNV Kunjin, are restricted in *P*. *leucopus* cells *in vitro* and that LGTV is a useful prototypic TBFV to further study interactions between these viruses and a natural host at the cellular level.

### Virus entry is not inhibited in *P*. *leucopus cells*

To understand how flaviviruses are restricted in *P*. *leucopus* cells, we evaluated specific steps (virus entry, protein translation, and RNA genome replication) of the virus life cycle. To determine if virus restriction in *P*. *leucopus* occurs through a block at the virus entry step or due to a lack of virus receptors we used a virus entry assay. Briefly, cells plated in chamber slides were infected and inoculum was removed 1 h post infection. The cells were washed with acid buffer to remove all bound, but not endocytosed, virus, and then the cells were fixed and the number of infected cells were visualized (Fig 2B). A representative infected cell is presented at 20X and 60X magnification and clearly indicates the viral E protein localized to endosome-like structures, as reported previously for other flaviviruses [36]. There was no significant difference in the number of virus-positive *P*. *leucopus* cells compared to the *M*. *musculus* cells at any MOI tested (Fig 2B). To confirm the staining observed in Fig 2B was due to virus entry, we treated both cell types with bafilomycin A1 (BAF), an inhibitor of the endocytic trafficking pathway [37], and observed a reduced number of infected cells. Furthermore, the RNA population that is predominant at early stages of infection (positive sense RNA) was measured by RT-qPCR and there was no significant difference in the abundance of viral genomic RNA within *P*. *leucopus* and *M*. *musculus* cells immediately following infection at 2 hpi (Fig 2C). These data suggest that virus entry is similar for resistant and susceptible cells and therefore that restriction is not mediated by lack of a cellular receptor.

### Viral RNA is successfully translated in *P*. *leucopus*

After virus entry, the positive strand RNA of flaviviruses can immediately serve as messenger RNA and be translated to protein in the host cell [38]. The newly-synthesized viral proteins function in virus RNA replication, packaging of new virion particles, and antagonism of the host cell defense [39,40].

To test the ability of viral RNA to be translated in *P*. *leucopus* cells, we used an infectious clone construct available for LGTV studies [41] where the genomic sequence is downstream of a T7 promoter. We performed *in vitro* transcription (IVT) and transfected the full-length positive sense single stranded RNA into the fibroblasts, which bypasses a requirement for viral entry. Transfected cells were fixed and stained for virus E and NS3 proteins. We were able to detect LGTV protein staining in both the *P*. *leucopus* and *M*. *musculus* cells (Fig 2D), showing that viral RNA can be translated to protein in the reservoir host. This is in line with NS3 and E protein expression visualized in virus-infected cells shown in Fig 1A. These data suggest that viral RNA can be translated in P. leucopus cells and TBFV restriction does not occur at the translation stage.

### Replication of viral RNA is significantly inhibited in *P*. *leucopus* cells

**Fig 3:**
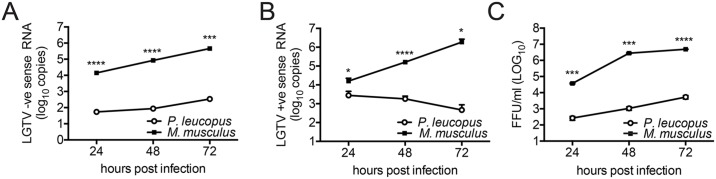
LGTV restriction in *P*. *leucopus* cells occurs at the replication phase. *P*. *leucopus* and C57BL/6 cells were infected at MOI 10. RNA lysates were collected at the indicated times post-infection and the resulting cDNA was used as template for RT-qPCR. The quantity of viral RNA was determined by the relative standard curve method to determine the abundance of: (A) LGTV negative strand (-) RNA from cell lysates (B) LGTV positive strand (+) RNA from from virus released into the cell culture supernatants and (C) LGTV focus-forming units (FFU/ml) titrated from virus recovered in the cell culture supernatant. Mean ± SD, data are from three independent experiments performed in triplicate. Asterisks indicate: * = p < 0.05, ** = p <0.01, *** = p <0.0001.

Despite no differences in virus entry, viral protein accumulation is reduced over time in *P*. *leucopus* cells (Fig 2A) suggesting that restriction may occur at the level of viral RNA replication. Positive strand RNA genome replication is initiated by synthesis of a complimentary negative strand, that then serves as a template for the synthesis of more positive strand copies. To determine whether LGTV genomic RNA is amplified in *P*. *leucopus* cells, we used RT-qPCR to measure the relative abundance of negative strand RNA. We observed significantly less negative strand RNA in *P*. *leucopus* cells compared to the control cells (Fig 3A), marking the first stage of the TBFV life cycle that is convincingly impaired in *P*. *leucopus* cells. This reduced RNA virus replication was associated with reduced production of secreted virions as measured by quantitation of total secreted positive strand genomic RNA by RT-qPCR (Fig 3B) and titration of infectious virions (Fig 3C). Taken together, these data suggest that LGTV can infect *P*. *leucopus* cells, but host cell-specific restriction suppresses RNA replication and subsequent production of infectious virus.

### Restriction of LGTV replication is associated with intrinsic cellular responses in *P*. *leucopus*

We next wanted to determine whether the failure of TBFV to replicate was due to a lack of necessary host factors or due to active suppression by a host antiviral response. A basic side-by-side comparison of general host responses for restricted and susceptible cells has not been completed for TBFVs to our knowledge. The trade-off for using a new model system is that few research reagents are available. To address our questions in the absence of specific *P*. *leucopus* reagents, we identified a panel of murine antibodies available for *M*. *musculus* that recognize *P*. *leucopus* proteins, including three IFN stimulated genes (ISGs): signal transducer and activator of transcription 1 (STAT1), IFN-induced protein with tetratricopeptide repeats 2 (IFIT2) and IFIT3. Unfortunately, we were unable to identify an antibody to recognize IFN alpha receptor 1 (IFNAR1) or IFNAR2. Additionally, due to lack of gene sequence we were unable to clone the IFN-β gene from *P*. *leucopus* and thus used commercially available murine IFN-beta (mIFN-β) for our studies. Importantly, treatment of *P*. *leucopus* cells with mouse IFN induced an antiviral state that inhibited VSV and further restricted LGTV (data not shown), suggesting that this reagent could be used in the context of *P*. *leucopus* cells.

**Fig 4:**
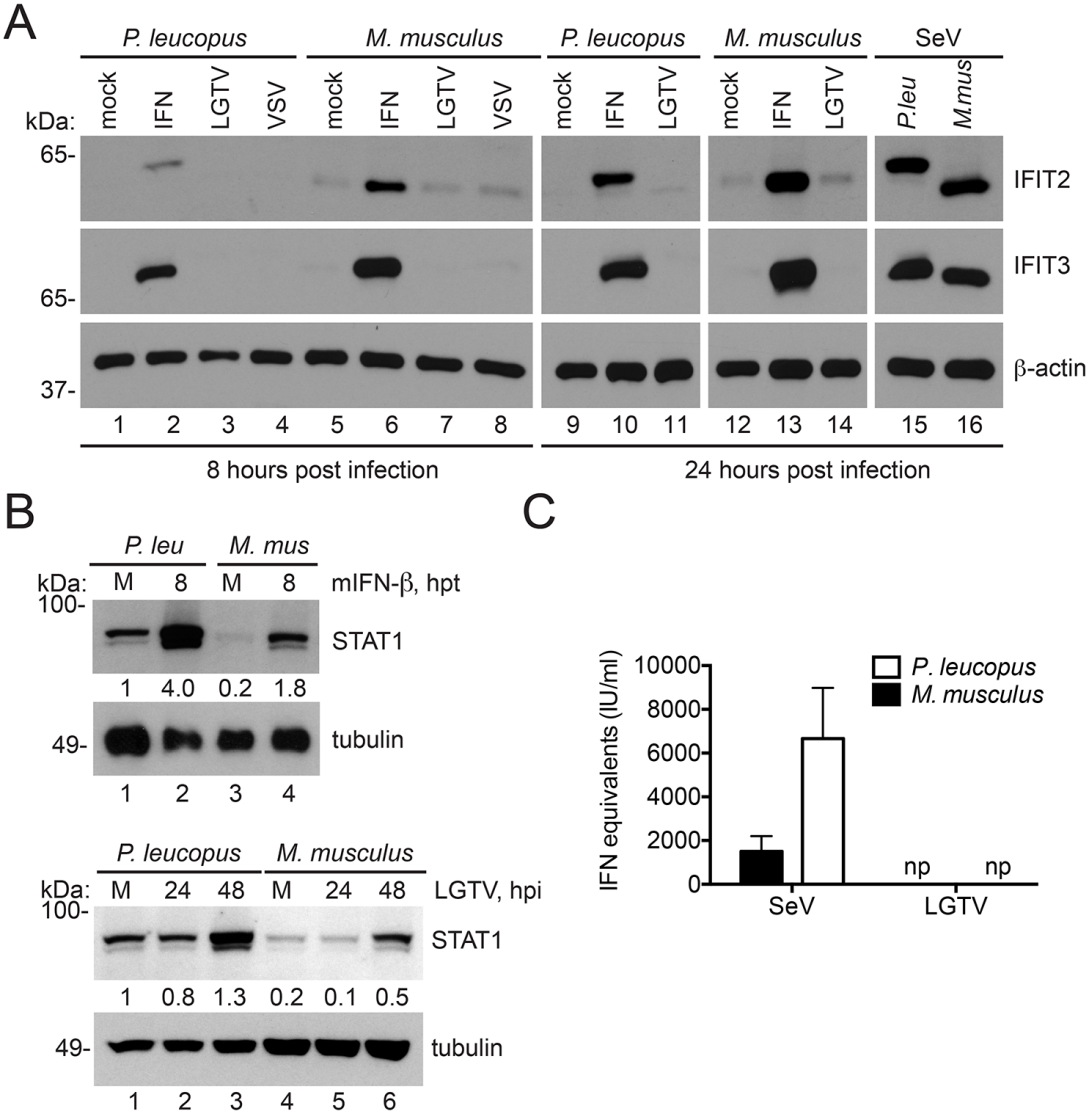
IFN stimulated proteins are expressed in *P*. *leucopus* during LGTV infection. Expression of ISGs in the reservoir host are shown in (A) Immunoblot of IFIT2 and IFIT3 expressed in *P*. *leucopus* and *M*. *musculus* cells challenged with mIFN-B, LGTV, VSV or SeV for 8 h or 24 h respectively. (B) Immunoblot of STAT1 expressed in *P*. *leucopus* and *M*. *musculus* cells challenged with mIFN-β (8 h) and LGTV (24 h and 48 h). Quantitation of STAT1 expression normalized to tubulin and relative to lane 1 is presented below STAT1 panels (C) Quantitation of antiviral assay performed in *P*. *leucopus* and *M*. *musculus* cells pre-treated with supernatants from corresponding cells that were previously infected with SeV or LGTV. An IFN-β standard curve in the respective species was used to calculate protection from individual samples. Mean ± SD, data are representative from three independent experiments. Hours post infection (hpi), hours post treatment (hpt), no protection (np).

To study antiviral responses, we first compared general ISG protein expression in *P*. *leucopus* and *M*. *musculus* cells. Not surprisingly, treatment of *P*. *leucopus* cells with mIFN-β resulted in the robust expression of IFIT2, and IFIT3 (Fig 4A) as well as STAT1 (Fig 4B). While STAT1 protein migrates at a similar molecular weight in both *P*. *leucopus* and *M*. *musculus* cells, we observed slightly smaller IFIT2 and IFIT3 proteins in *P*. *leucopus* cells, raising the possibility that the antiviral response may be substantially different between the two rodent species.

Unlike IFIT2 and IFIT3, both the basal and IFN-induced levels of STAT1 (Fig 4B) were elevated in *P*. *leucopus* cells. A similar increased induction of STAT1 was observed during LGTV infection (Fig 4B). STAT1 is a transcription factor that is necessary for IFN signal transduction [42,43]. Thus, increased STAT1 expression could indicate that *P*. *leucopus* cells express higher levels of IFN, are more responsive to IFN, or LGTV lacks the ability to prevent antiviral gene expression. To test whether *P*. *leucopus* cells express high levels of IFN, we infected *P*. *leucopus* and *M*. *musculus* cells with either LGTV or Sendai virus (SeV), harvested supernatants, and measured the ability of the supernatant to protect corresponding fresh *P*. *leucopus* and *M*. *musculus* cells from VSV infection. No protection was obtained from supernatants taken from mock-infected *M*. *musculus* or *P*. *leucopus* cells. Supernatants from SeV-infected cells were effective in inducing an antiviral state, and *P*. *leucopus* supernatants were 4 times more protective than *M*. *musculus* supernatants (Fig 4C). However, supernatants from LGTV-infected cells were incapable of protecting fresh fibroblasts from VSV infection consistent with the observation that flaviviruses induce very little IFN during infection [44]. The data suggest that *P*. *leucopus* fibroblasts can produce substantial levels of IFN in response to a prototype virus (SeV), but in the presence of LGTV, little IFN is induced and restriction is not associated with high basal IFN secretion. Therefore, if IFN signaling is necessary for TBFV restriction, it is likely through the up-regulation of specific antiviral genes and not due to accumulation of IFN to high concentrations.

### Homologs of antiviral signaling genes are expressed in *P*. *leucopus* and upregulated by IFN treatment and virus infection

While viral recognition and antiviral signaling factors have been widely studied in various species, little is known about the expression of those genes in *P*. *leucopus* and how they function in response to infection. We identified and sequenced homologs of important signaling proteins in *P*. *leucopus* involved in cytosolic responses to RNA viruses including retinoic acid inducible gene I (RIG-I), mitochondrial antiviral signaling protein (MAVS), IFN regulatory factor 1 (IRF1), STAT1 and IFNAR1. Additionally, we identified a homolog of the only known TBFV-specific restriction factor TRIM79 (also known as TRIM30D, NCBI Gene ID#209387) belonging to the tripartite motif family of proteins [45]. The gene sequence for these *P*. *leucopus* genes are relatively similar to their counterparts in *M*. *musculus* (Table 1).

**Table 1:** Comparison of *P*. *leucopus* gene homologs to *M*. *musculus* genes.

*P*. *leucopus* gene	*P*. *leucopus* accession number	% Identity	*M*. *musculus* accession number
STAT1	KY451962	90%	NM_001205313.1
IFNAR1	KY451965	78%	NM_010508.2
RIG-I	KY451966	88%	NM_172689.3
MAVS	KY451963	82%	NM_144888.2
IRF1	KY451964	89%	NM_008390.2
TRIM79	KY451967	75%	XM_006507549.2

Summary of alignment data showing the percentage identity of newly resolved *P*. *leucopus* gene homologs to their *M*. *musculus* counterparts. Accession numbers of the gene references are shown on the far right of the table.

**Fig 5:**
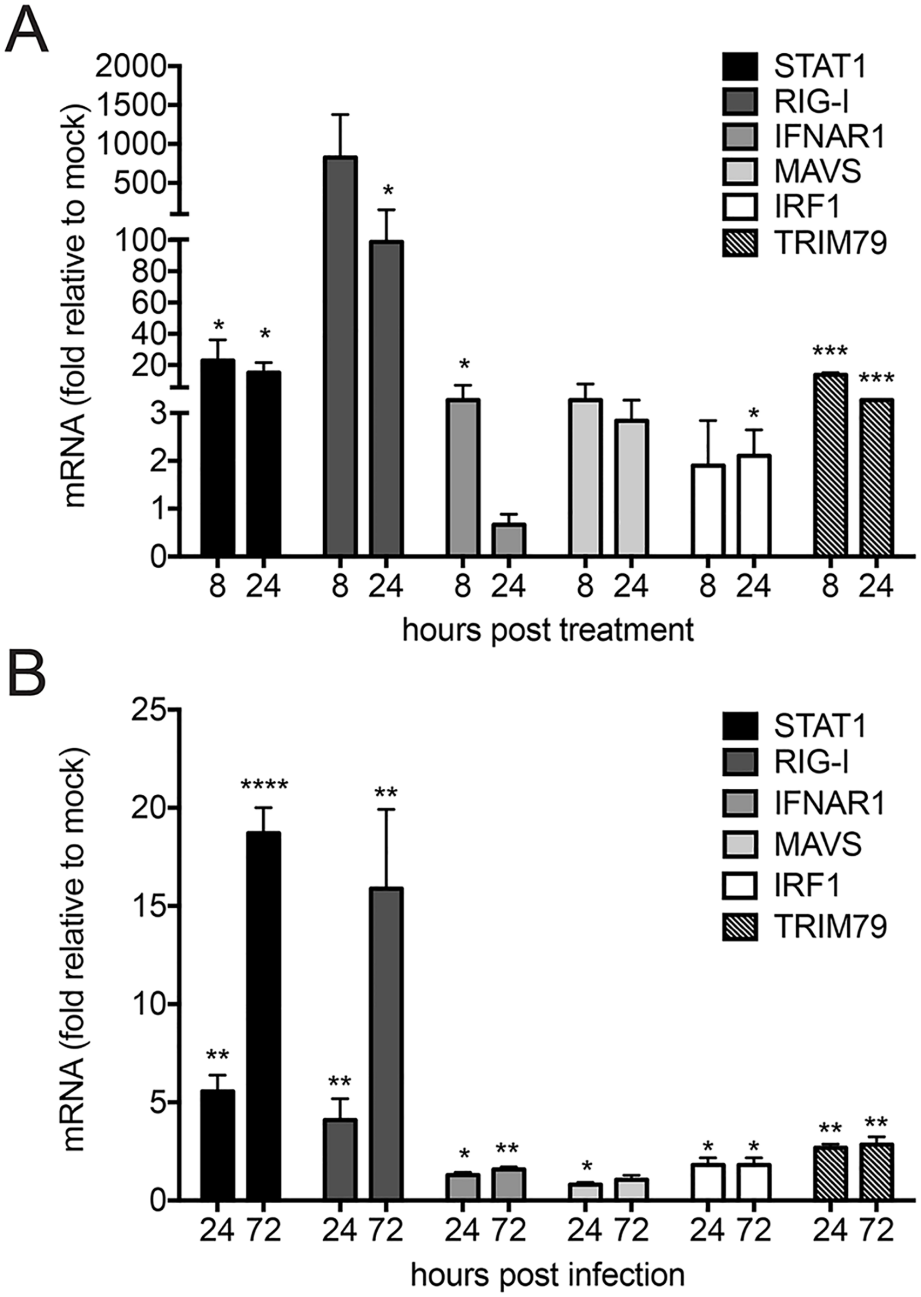
Kinetics of antiviral signaling homologs in *P*. *leucopus* assessed with novel sequence information. RT-qPCR showing relative fold induction of STAT1, RIG-I, IFNAR1, MAVS, IRF-1 and TRIM79 in *P*. *leucopus* cells that were (A) treated with mIFN-β for 8 h and 24 h or (B) infected with LGTV for 24 h and 72 h respectively. Cellular cDNA was probed using gene-specific primers designed from gene sequences in *P*. *leucopus*. Data were quantified relative to mock-treated/infected cells and normalized to beta-actin levels and are representative of three independent experiments performed in triplicate. Asterisks indicate: * = p < 0.05, ** = p <0.01, *** = p <0.0001.

To understand how *P*. *leucopus* cells respond to IFN and virus infection, we examined antiviral gene expression by RT-qPCR using primers based on the *P*. *leucopus* gene sequences. As expected, the known ISGs STAT1, RIG-I, and TRIM79 were upregulated greater than 10 fold in response to IFN treatment, whereas increases in IFNAR1, MAVS, and IRF1 levels were modest (Fig 5A). It is interesting to note here that in susceptible cells—both human and *M*. *musculus*, IRF1 is potently induced by IFN and is a critical component to the antiviral state to flaviviruses [46,47]. Consistent with the weaker protein responses observed by western blot (Fig 4), LGTV infection was a weaker stimulus for *P*. *leucopus* antiviral gene expression when compared to IFN. However, induction followed the same pattern seen in IFN treatment as STAT1, RIG-I, and TRIM79 were all upregulated at least 3 fold (Fig 5B). Thus infection with LGTV induces an ISG transcriptional response in *P*. *leucopus* cells.

### LGTV NS5 protein antagonizes IFN signaling in *P*. *leucopus cells*

Since antiviral genes are expressed during LGTV infection of *P*. *leucopus* cells, we questioned whether TBFVs are capable of antagonizing IFN-induced transcriptional responses in *P*. *leucopus* cells. IFN antagonism is crucial for efficient flavivirus replication and has a significant impact on the outcome of infection, for both *in vitro* and in *in vivo* models [48,49]. We and others have shown that the NS5 protein from both tick-borne and mosquito-borne flaviviruses functions as an antagonist of type I IFN signaling by preventing IFN-induced JAK-STAT signaling to suppress ISG expression and establishment of an antiviral state [50–52].

**Fig 6:**
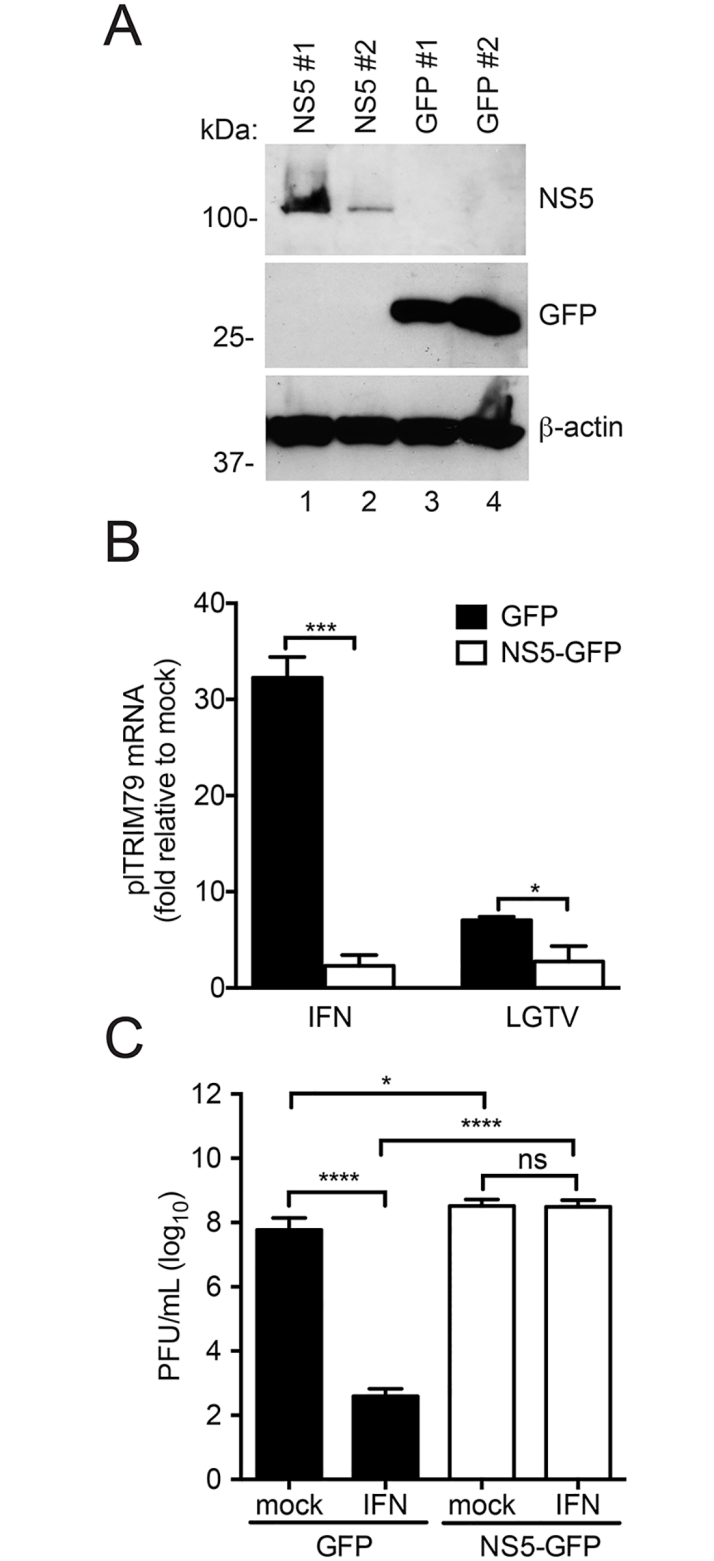
LGTV NS5 functions as an IFN antagonist in *P*. *leucopus* cells. Cell lines were generated in *P*. *leucopus* to test NS5 functionality. (A) Immunoblot showing overexpression of NS5. *P*. *leucopus* fibroblasts were transduced with lentiviruses expressing LGTV NS5–GFP fusion or GFP vector only. (B) RT-qPCR showing relative expression of an ISG (plTRIM79) in the presence of LGTV NS5. Cells were treated with mIFN-β (8 h) or infected with LGTV (24 h). Expression of plTRIM79 was assessed by RT-qPCR using gene-specific primers. (C) Antiviral assay using VSV in *P*. *leucopus* cells overexpressing LGTV NS5. Cells were pretreated (or not) with mIFN-β for 16 h prior to infection with VSV for an additional 24 h. Supernatants from virus-infected cells were titrated by a plaque assay. Mean ± SD, data are from three independent experiments performed in triplicate. Asterisks indicate: * = p < 0.05, ** = p <0.01, *** = p <0.0001.

To test if NS5 can antagonize IFN signaling in *P*. *leucopus* cells, we constructed clonal *P*. *leucopus* cell lines to over-express either green fluorescent protein (GFP) alone or fused with LGTV NS5 (Fig 6A). The transcriptional response to IFN was measured by examining *P*. *leucopus* (pl)lTRIM79 mRNA induction as a representative ISG. NS5-GFP expressing *P*. *leucopus* cells failed to express TRIM79 mRNA in response to mIFN-β treatment (Fig 6B), SeV (data not shown), and LGTV infection (Fig 6B), suggesting that NS5 can interfere with IFN signal transduction in *P*. *leucopus* cells regardless of tested stimulus. NS5 expression also prevented the establishment of an antiviral state in IFN-treated cells as measured by VSV replication in GFP- or NS5-expressing cells (Fig 6C). Collectively, these data demonstrate that the viral NS5 protein functions as an IFN antagonist in *P*. *leucopus* cells. Thus, a lack of IFN-antagonism by the virus in *P*. *leucopus* cells is unlikely to contribute to the restricted replication of TBFV in this host.

### Type I IFN signaling is necessary for LGTV restriction in *P*. *leucopus* cells

**Fig 7:**
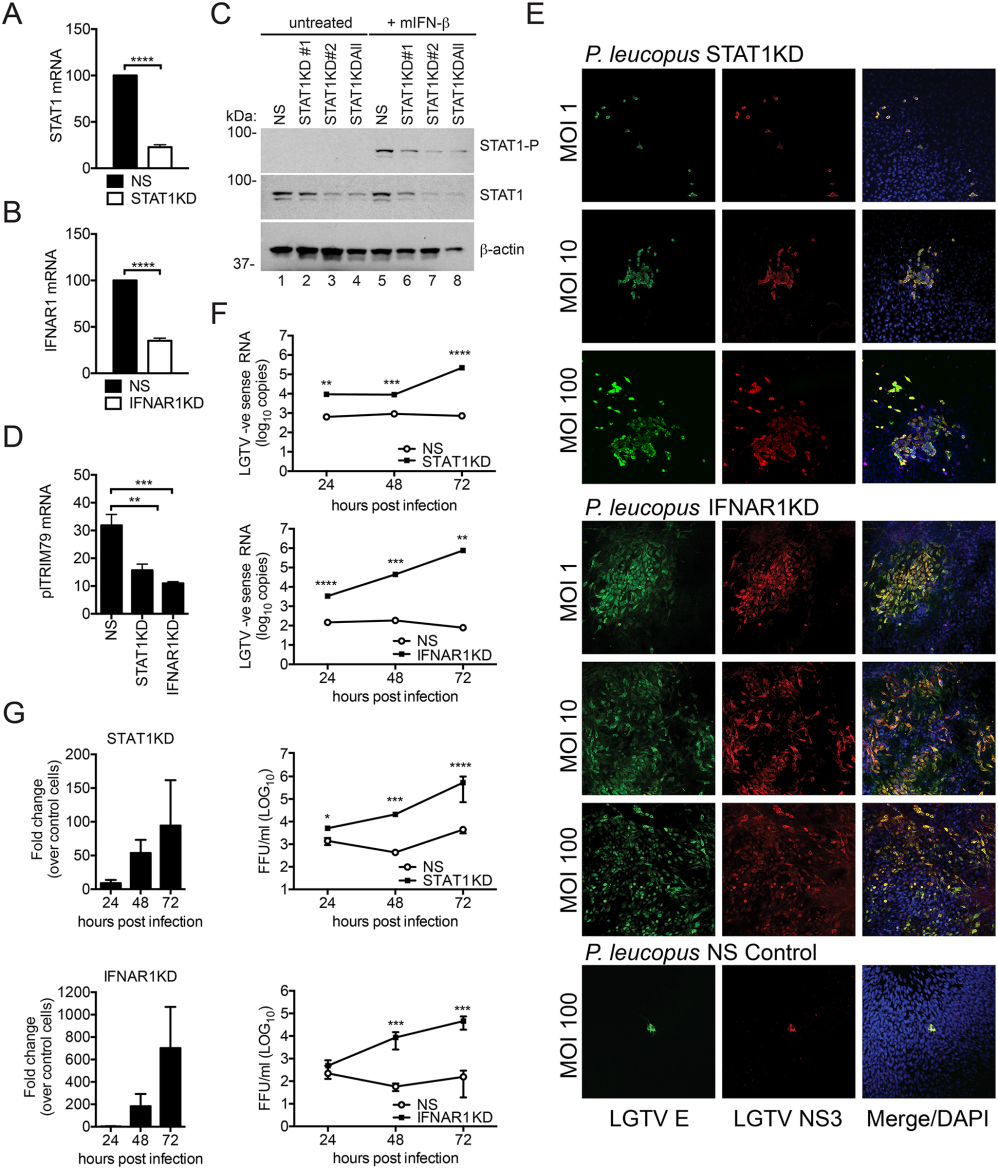
IFN signaling in *P*. *leucopus* is critical for the virus restriction phenotype. Newly-resolved sequence of STAT1 (A) and IFNAR1 (B) were targeted by shRNA technology. Cell lines were generated by lentiviral transduction and cell lysates were assessed for knockdown by qPCR using gene-specific primers. Data are shown as percentage gene expression relative to the NS control. (C) Immunoblot showing expression of STAT1 and STAT1-P in knockdown cells targeted with various short hairpins (#1, #2 and ALL) compared to the non-silencing (NS) control. Cells were treated with mIFN-β for 8 h before cell lysates were harvested. (D) RT-qPCR of ISG plTRIM79 in STAT1KD, IFNAR1KD, and NS control cells treated with mIFN-β. Data are shown as relative fold induction of plTRIM79 compared to mock-treated cells. (E) STAT1KD cells, IFNAR1KD, and control (NS) cells of *P*. *leucopus* were infected with LGTV for 72 h and immunostained for the viral E protein (green), NS3 protein (red), and nuclei were stained with DAPI (blue). Cells were visualized by confocal microscopy (20X magnification). (F) STAT1KD cells (top panel) and IFNAR1KD cells (lower panel) of *P*. *leucopus* were infected with LGTV at MOI 10. Cell lysates were collected at the indicated time points post-infection and probed for LGTV negative strand (-) RNA by RT-qPCR (as a replication marker). Viral RNA abundance was determined by a standard curve method and compared to the NS control. (G) STAT1KD cells (top panels) and IFNAR1KD cells (lower panels) of *P*. *leucopus* were infected with LGTV at an MOI 10. Viral supernatants were collected at the indicated time points and titrated by an immunofocus assay compared to supernatant from the NS control cells. Data presented as fold change relative to NS cells (left panels) or as quantitated virions (right panels). Mean ± SD, Data are from three independent experiments performed in triplicate. Asterisks indicate: ** = p<0.01, *** = p<0.001, **** = p<0.00001.

To determine whether the IFN response contributes to specific virus restriction in *P*. *leucopus* cells, we designed and delivered short hairpin RNA (shRNA) into *P*. *leucopus* cells to knockdown expression of either STAT1 or IFNAR1. Knockdown of STAT1 (STAT1KD, Fig 7A) or IFNAR1 (IFNAR1KD, Fig 7B) compared to non-silencing (NS) control cells was confirmed at the mRNA level, as well as at the protein level for STAT1 (Fig 7C). Knockdown of either STAT1 or IFNAR1 resulted in reduced induction of plTRIM79 mRNA following stimulation with IFN (Fig 7D). We next infected the *P*. *leucopus* STAT1KD, IFNAR1KD, or NS cells with LGTV and examined the infected cells by confocal microscopy using virus-specific antibodies. Markedly more infected cells were apparent when STAT1 or IFNAR1 were knocked down, while the control NS cells were resistant to infection similar to unmodified *P*. *leucopus* cells (Fig 7E). Of note, the staining at MOI 1 in the IFNAR1KD was similar to what we observed with *M*. *musculus* cells (compare to Fig 1A). The increased replication of LGTV was confirmed by both negative sense RNA accumulation measured by RT-qPCR (Fig 7F) and a significant increase in infectious virus release, that was up to 94.4 and 701.2 fold higher than virus released from control cells in STAT1KD and IFNAR1KD cells, respectively (Fig 7G). Thus, the restriction observed in *P*. *leucopus* cells is strongly influenced by the intrinsic cellular response to infection. The high level of restriction observed was dependent on type I IFN signaling through IFNAR1 and STAT1 despite what must be a very limited amount of IFN produced and a robust ability of the virus to antagonize IFN-dependent signaling in infected cells.

## Discussion

Reservoir species are necessary to maintain a minimum infective presence of pathogens in nature [4,22]. However, it is not understood whether the pathogens remain undetected by the reservoir host immune response or if the host responds to infection with a potent immune response to maintain low levels of virus replication and prevent disease. Using the model of the white footed mouse, *P*. *leucopus*, and TBFVs, we have shown that replication of LGTV, POWV, or TBEV in primary fibroblasts occurs only at very low levels as compared to replication in fibroblasts from permissive C57BL/6 mice. The restriction of TBFVs was linked to the intrinsic cellular response to infection, as it was dependent on signaling through both IFNAR1 and STAT1. However, it is noteworthy that the innate response that limited replication of LGTV did not function to restrict replication of a highly IFN-sensitive virus, VSV. The *P*. *leucopus* cells were competent for IFN expression, but did not produce large amounts of IFN in response to virus infection. Instead, we observed high basal expression of STAT1 (see quantitation of Fig 4A and 4B, mock-treated *M*. *musculus* cells consistently expressed less total STAT1 protein than *P*. *leucopus* cells.) that might indicate that the *P*. *leucopus* cells have an increased response to IFN compared to the permissive C57BL/6 cells. A similar phenomenon is thought to protect cardiomyocytes against virus infection [53]. However, if this were the case, we would expect to observe greater restriction of VSV replication in *P*. *leucopus* cells. Alternatively, the ISGs produced in response to IFN may exhibit specificity towards certain pathogens including the TBFVs.

The *in vitro* phenotype recapitulated in fibroblasts provided a model system to systematically compare the virus infection cycle in the resistant and susceptible cells to identify the specific step that is blocked during virus infection of reservoir host cells. Fibroblasts were used in these studies as they are among the initial cells to become infected in the skin following the bite of an infected tick, and they also proved to be a valuable workhorse for making stable cell lines to probe molecular factors influencing virus replication. At early stages of infection, there is relatively equal amount of virus RNA detected in both the resistant and susceptible cells suggesting that the virus can gain entry into *P*. *leucopus* cells efficiently. This suggests that the cells are permissive to virus infection at higher doses, thus supporting the notion that virus can overcome restriction by certain ISGs as the antiviral effects of ISGs are saturable [47].

We have shown that the NS5 protein of LGTV functions as an efficient antagonist of IFN signaling in *P*. *leucopus* cells (Fig 6). Previous studies have highlighted the importance of viral antagonism to host species tropism of flaviviruses. NS5 from both DENV and ZIKV degrades STAT2 in human or non-human primate cells but not in mice which likely contributes to resistance of immunocompetent mice to infection with these viruses [54–56]. Therefore, any variation in the ability of LGTV NS5 to antagonize IFN signaling in *P*. *leucopus* cells could explain the resistance observed in the reservoir cells as compared to susceptible host cells. However, this was not observed. It is therefore remarkable that *P*. *leucopus* cells could mount a strong antiviral response against LGTV in the presence of a potent viral IFN antagonist that should provide a significant degree of resistance. It is possible that lower viral titers in reservoir host cells are due to a more potent counter response to viral antagonism thus overriding NS5 activity during infection. Although not completely understood, this phenomenon points to the evolutionary arms race occurring between pathogens and hosts wherein a pathogen develops a means to evade host responses and the host in turn counteracts the evasion mechanism [57]. Detailed studies are needed to clearly understand the role of NS5 or other mechanisms of IFN antagonism during TBFV infection of *P*. *leucopus*.

This model is a starting point for further identification of reservoir host factors responsible for specific inhibition of virus infection. Importantly, we demonstrated that the resistance phenotype is likely due to active suppression of virus replication in *P*. *leucopus* by an antiviral signaling cascade and therefore excluded the option that low virus titers are due to the lack of a proviral factor. Higher virus replication in the IFNAR1KD and STAT1KD cells (Fig 7) suggests that an antiviral response rather than a lack of proviral factors accounts for reservoir host resistance. Moreover, restriction in *P*. *leucopus* cells is successively overcome by an increase in the MOI (Fig 1B) suggesting that the virus is able to enter and replicate in these cells but is simply maintained at low levels. The observation that IFN signaling regulates TBFV infection is not particularly surprising as the role of the IFN response to viruses has been well established [58,59]. However, the novelty in our study lies in demonstrating the role of IFN signaling in a reservoir species *P*. *leucopus* which is inherently resistant to TBFV infection. Disease tolerance and resistance are two concepts that could apply to a host that remains disease-free despite infection [17,60]. Tolerance is a passive ability of the host to withstand the harmful effects of infection whereas resistance is an active process of suppressing infection to prevent disease [4,60]. Findings from our STAT1 and IFNAR1 knockdown studies point to an active resistance in *P*. *leucopus* and can form the basis for further probe into the immune system of natural reservoir hosts.

IFN signaling ultimately results in the upregulation of ISGs. These include numerous host factors that could elicit a broad or specific effect to curb virus replication and prevent the spread of infection [59,61]. Since VSV replication is not restricted (Fig 1B), we propose that flaviviruses are specifically targeted. Indeed, co-evolution of TBFVs with *P*. *leucopus* has likely resulted in the development of potent IFN-stimulated antiviral factors with potential broad-reaching impacts on other closely-related flaviviruses that have not been shown to co-evolve with this reservoir host as exemplified by the restriction of WNV Kunjin shown in Fig 1G. Restriction factors (RFs) have been found to act at various stages of infection and our data suggest that the TBFV RFs in *P*. *leucopus* are likely acting at the replication stage potentially leading to less accumulation of viral RNA available for packaging and consequently, less production of new virion particles. Flavivirus replication is a complex process that requires the viral enzymes NS3 and NS5 [1]. RFs could function indirectly by affecting host cellular processes necessary for virus replication or could act more directly by binding to viral proteins and preventing their functionality. We previously described the only known TBFV-specific RF TRIM79 in a murine system [45]. TRIM79 is an ISG that binds to NS5 and targets it for lysosome-mediated degradation, thereby leading to lower virus replication [45]. In the current study, we found that *P*. *leucopus* cells express similar TRIM proteins during TBFV infection, including the *P*. *leucopus* homolog of TRIM79 (plTRIM79). Additionally, we found that despite only 75% protein sequence conservation with the *M*. *musculus* TRIM79, plTRIM79 also interacted with NS5 (data not shown). Studies are currently underway to evaluate the importance of plTRIM79 and other *P*. *leucopus* genes as potential TBFV RFs.

Based on the common ancestry relationship between *P*. *leucopus* and *M*. *musculus*, it is expected that similar genes will be expressed and homologs of RF candidates will be identified in the reservoir species. However, we hypothesize that differences in the gene sequences could potentially result in variations in binding sites, binding partners, post-translational modifications, relaxed or narrowed specificity, and expression patterns of the gene homologs in *P*. *leucopus*. These genetic differences could thus be sufficient to protect the host and launch a balanced antiviral response that prevents disease development while infection remains at low levels. On the other hand, RFs in the susceptible species likely lack certain binding sites and/or partners resulting in a relatively modest host response and disease development. Indeed, a more detailed study and comparison of specific RFs (such as TRIMs, IFITs, and oligoadenylate synthetase (OAS) [62–64] genes) in *P*. *leucopus* will provide information on any genetic variations and help us narrow down resistance determinant to specific genetic loci. Ultimately, RFs identified in cell culture models could be tested *in vivo* to determine their role during TBFV infection and neurotropism of the whole organism.

Overall, our study describes a novel model system of virus restriction in the natural reservoir host *P*. *leucopus*, identifies the restriction point in the life-cycle to be at RNA replication, identifies new antiviral gene homologs in the reservoir species, and demonstrated that restriction is due to an antiviral response through the IFN response pathway. Studies are underway to identify specific RFs that interact with viral proteins in *P*. *leucopus* cells to suppress virus replication.

## Materials and methods

### Cell culture and reagents

Human embryonic kidney (HEK) 293, HEK 293T, Vero, *P*. *leucopus* adult skin fibroblast cells (AG22353, *Peromyscus* Genetic Stock Center (PCSC), University of South Carolina) and *M*. *musculus* (C57BL/6) murine embryonic fibroblast (MEF) cells [45] were grown in Dulbecco’s modified enrichment medium (DMEM) containing 10% (vol/vol) fetal bovine serum (FBS, Gibco) 100 units/ml penicillin, and 100 mg/ml streptomycin (Thermo Fisher Scientific) in an atmosphere of 5% CO2 at 37°C. *Peromyscus* embryonic fibroblasts (PEFs) were generated using our previous described protocol [45]. Tissues for cell isolation were purchased from the PCSC and produced under IACUC protocol #2162-100829-0.

### Plasmids and vectors

Entry vectors used in this study were pENTR-D-TOPO (Thermo Fisher Scientific) and pENTR-U6 (Thermo Fisher Scientific). Langat virus (LGTV) nonstructural (NS) protein 5 (NS5) was derived by PCR amplification using the LGTV E5 infectious cDNA clone as template (provided by Dr. A. Pletnev, NIAID, NIH) as previously described [65]. The PCR product was directionally cloned into the entry vector by gateway cloning and further recombined into the lentiviral expression plasmid pLVU-GFP (Addgene #24177) with the vector backbone pLenti6/UbC/V5-Dest. To design lentiviral plasmids for gene knockdown, plenti-X2-hygro-DEST (Thermo Fisher Scientific) was used as a destination vector.

### Antibodies

The following antibodies were used: α-actin (A5441, Sigma); α-tubulin (sc-12462, Santa Cruz); α-GFP (JL-8, 632381, Clontech); α-dsRed (632496, Clontech); α-V5 (R960-25, Invitrogen); α-STAT1 (9172S, Cell Signaling Technology); α-STAT1-P (9167S, Cell Signaling Technology); α-LGTV E and NS1 (provided by Dr. C. Schmaljohn, USAMRIID); α-LGTV NS3 [45]; α-IFIT2 and α-IFIT3 (provided by Dr. S. Chattopadhyay, University of Toledo).

### Viruses and infections

The following viruses were used in this study: LGTV (TP21 strain, provided by Dr. A. Pletnev, NIAID, NIH), vesicular stomatitis virus (VSV, strain Indiana, provided by Dr. I. Novella, University of Toledo), tick-borne encephalitis virus (TBEV, strain Sofjin, provided by Dr. M. Holbrook, NIAID, NIH), POWV lineage I (POWV-I, strain LB) and POWV lineage II (POWV-II, strain Spooner), and WNV (Kunjin strain) provided by Dr. S. M. Best, NIAID, NIH, and Sendai virus (SeV, strain Cantell; Charles River Laboratories). Virus working stocks were propagated and titrated by immunofocus assay (LGTV) or plaque assay (TBEV, POWV, and VSV) on Vero cells. All procedures with POWV were performed under biosafety level-3 (BSL-3) conditions; procedures with TBEV were performed under BSL-4 conditions at the Rocky Mountain Laboratories Integrated Research Facility (Hamilton, MT). Multiplicity of infection (MOI) is represented as focus forming units (FFU) or plaque forming units (PFU) per cell.

### Virus titration by immunofocus assay and plaque assay

For flavivirus quantitation, test cells were set up at 1 X 10^5^ per well in 24-well dishes and infected with the indicated MOI. Viral supernatant was collected at various time points post infection. The virus was titrated by performing 10-fold dilutions of the supernatants and infecting Vero cells (2 X 10^5^/well) with 250 μl of diluted virus stocks. After 1h adsorption period, the inoculum was removed and the cells were overlaid with growth medium containing 0.8% methylcellulose (w/v) and 2% (vol/vol) FBS (Gibco). At 4-days post infection, the infected Vero cells were washed twice with PBS and fixed with 100% methanol for 30 min. at room temperature (RT). Plates were washed twice with PBS and then blocked with OptiMEM for 30 min. at RT. Cells were then incubated with virus-specific antibodies (α-LGTV E for LGTV) for 1 h at 37°C. The plates were washed twice with PBS and incubated with horseradish peroxidase (HRP)-conjugated secondary antibodies (Dako) in OptiMEM for 1 h at 37°C. Cells were washed twice with PBS and the FFU were visualized with freshly prepared peroxidase solution containing 0.4 mg/ml 3,3’-diaminobenzidine (Sigma) and 0.0135% hydrogen peroxide in PBS. Plaque assays to quantitate VSV were performed as with the immunofocus assays with the exception that following a 48 h incubation, the cells were washed twice in PBS and fixed with crystal violet (0.8% in ethanol). To quantitate POWV and TBEV, modified plaque assays were performed using 1.5% carboxymethylcellulose sodium salt (Sigma Aldrich) as an overlay and plates were fixed at 4 days post-titration with 10% formaldehyde and further stained with crystal violet (0.8% in ethanol)

### Immunofluorescence confocal microscopy

Cells were plated in Lab-Tek II chamber slides (Thermo Fisher Scientific) and prepared by washing twice with PBS before fixing with 4% paraformaldehyde for 20 min. Cells were washed 3 times with PBST (PBS, 0.5% Tween-20) and then incubated with permeabilization buffer (0.1% Triton X-100, 0.1% sodium citrate) for 5 min. followed by incubation with blocking solution (PBS, 0.5% BSA, 1% goat serum) for 1 h at RT. Cells were then incubated with primary antibody for 1 h at RT, washed 3 times with PBST and incubated with secondary antibodies conjugated to Alexa-488 or Alexa-594 (Thermo Fisher Scientific) for 1 h. Slides were washed 3 times with PBST in the dark and overlaid with glass coverslips using Prolong Gold + DAPI mounting media (Thermo Fisher Scientific). Stained cells were visualized using an Olympus confocal microscope (Olympus Fluoview FV1000).

### Western blotting

Cells were washed twice in PBS and lysed in radioimmunoprecipitation assay (RIPA) buffer (50 mM Tris-HCl, 150 mM NaCl, 0.1% SDS, 1% NP-40, 0.5% Na-deoxycholate) with complete protease inhibitor cocktail (Roche). Cell lysates were treated with Turbo DNase (Thermo Fisher Scientific) and cellular debris was removed by centrifugation (2,000 x *g* for 5 min) while the supernatant was reserved. Equal amounts (10–30 μg) of protein were loaded on a 10% polyacrylamide gel and resolved by electrophoresis. Protein was transferred on to a nitrocellulose membrane using the iBlot 2 Gel Transfer Device (Thermo Fisher Scientific). Membranes were probed with specific primary antibodies overnight at 4°C followed by a secondary incubation with goat anti-mouse IgG or goat anti-rabbit IgG antibodies (Thermo Fisher Scientific) for 1 h at RT. Immunoreactive proteins were detected using ECL Plus Western chemiluminescent system (Thermo Fisher Scientific) and exposed to film. Blot quantitation was performed using Image J software.

### *P*. *leucopus* gene sequencing

A search for the genes of interest was performed from a RNAseq database of *P*. *leucopus* cells from Dr. J. Munshi-South [66]. All the hits obtained for each gene sequence were aligned with the sequence of the corresponding *P*. *maniculatus* gene (STAT1-XM_006974860, RIGI-I-XM_006975324, IFNAR1-XM_006983579, MAVS-XM_006984557, IRF1-XM_006995899, TRIM79-XM_006507549.2). The consensus sequence from each alignment was used as a template for the design of cloning primers. Further, cDNA was purified from *P*. *leucopus* cells and probed using the gene-specific primers (available upon request). The PCR product was resolved on an agarose gel and further cloned into the entry vector pENTR-D-TOPO. DNA sequencing was performed on entry clones and PCR products using internal cloning and sequencing primers for complete resolution.

### Quantitative reverse transcriptase polymerase chain reaction (RT-qPCR)

Total RNA was purified from cultured cells using the RNeasy Mini kit (Qiagen) with DNase 1 digestion step (Qiagen). Resultant RNA was reversed transcribed using the QuantiTect reverse transcription kit (Qiagen). The cDNA was used as a template for SYBR green-based qPCR using the FastStart essential DNA GreenMaster (Roche) according to the manufacturer’s protocol. IFNAR1 Fwd–CTGGAGACCACTCGGATAAATG, Rev- CTCGTACCCGGAGAAAGAAAG; STAT1 Fwd–GAGAGAAACTTCTGGGTCCTAAC, Rev–GATCCAAGGCCAGAAGGAAA; MAVS Fwd- GTCTTCCTCTTCCACTGGATTG, Rev–GTCACAGAATTGGTGGGTACTT; RIG-I Fwd–GGTTCTGAAACTTGCTTTGGAG, Rev—GCAGCTTTACTTTCAACCCTTT; IRF1 Fwd–CAGCACCAGCGATCTGTATAA, Rev–TTCCTTCCTCGTCCTCATCT; β-actin (Fwd-CACACTGTGCCATCTATGA, Rev- GGATCTTCATGAGGTAGTCTGTC). All reactions were performed in triplicate in the Roche LightCycler^®^ 96 instrument and analyzed with the LightCycler^®^ 480 Software, Version 1.5. Results were normalized to mRNA levels of β-actin. For relative quantification in stimulated cells, results are expressed as a fold change relative to RNA samples from mock-infected, unstimulated cells using the comparative threshold cycle method. To assess viral RNA in virus-infected cells, absolute quantifications were performed using the relative standard curve method generated from 10-fold serial dilutions (10^9^−10^0^ genome copies) of the LGTV E5 infectious clone cDNA. Positive strand RNA of LGTV was assessed with primers previously described [67] using forward primer LGTV911F (GGATTGTTGCCCAGGATTCTC) and reverse primer LGTV991R (TTCCAGGTGGGTGCATCTC) and normalized to mRNA levels of β-actin from either *P*. *leucopus* or *M*. *musculus* (Fwd-GCAAGCAGGAGTACGATGAG, Rev-CCATGCCAATGTTGTCTT). Similarly, negative sense RNA of LGTV was quantified using forward primer-GTCTCCGGTTGCAGGACTGT and reverse primer–CTCGGTCAGTAGGATGGTGTTG [68]. To test viral RNA release after infection, viral supernatants were collected at various time points post infection. Viral RNA was purified from the supernatant using the QIAamp Viral RNA Mini Kit (Qiagen). The resultant RNA was normalized and used as template for cDNA synthesis. The cDNA obtained was further analyzed by qPCR using primers specific for the LGTV positive strand RNA as described above.

### Antiviral assay

The test cells were infected with LGTV (MOI 1 and 10) or SeV (600 hemagglutinin (HA) units) for the indicated period of time. Viral supernatant was collected and added to fresh cells in 18 2-fold serial dilutions. As a control, cells were treated with 18 2-fold dilutions of mouse interferon beta (mIFN-β, PBL Assay Science). Treated cells were infected at 16 h post-treatment with VSV (MOI 0.1). At 36 h post-infection, cells were washed twice with PBS and stained with crystal violet solution. Plates were evaluated by measuring the dilution at which 50% virus inhibition occurred and measured based on the mIFN-β standard dilution plate to extrapolate the level of IFN responsiveness in the test cells.

### Viral entry assays

Cells were plated in Lab-Tek II chamber slides (Thermo Fisher Scientific) or culture flasks at 1 X 10^5^ cells and infected with LGTV at varying MOIs. Cells were incubated at 4°C for 1 h and then shifted to 37°C for an additional hour. The cells were then washed 3 times in ice cold PBS followed by an acid glycine wash (137 mM NaCl, 5 mM KCl, 0.49 mM MgCl_2_.6H2O, 0.68 mM CaCl_2_.2H2O, 99.84 mM glycine, pH 2.0) to remove unbound virus. Slides were fixed and stained according to the immunofluorescence protocol. Assays were quantitated by visually counting the number of virus-positive cells stained with α-LGTV E. Cells grown in culture flasks were harvested for purification of total RNA and subsequent analysis by RT-qPCR.

### Infectious clone technology

Full-length plasmid DNA p61-E5 corresponding to the LGTV strain E5 (provided by Dr. A. Pletnev, NIAID, NIH) was digested for 4 h at 37°C. The linearized cDNA was cleaned up by incubating with 3M sodium acetate (NaCO_3_CO_2_) and 100% ethanol. Clean DNA pellet was recovered into solution and used as a template for *in vitro* transcription by incubating for 2 h at 40°C with the transcription cocktail (18.75 mM rATP, 18.75 mM rCTP, 18.75 mM rUTP, 3.75 mM rGTP, 15 mM m^7^G cap analog, SP6 polymerase, RNAse OUT, 100 mM DTT) and 1 μg of DNA. The resultant RNA was treated with DNAse to remove DNA contamination. Cells were transfected with viral RNA using Lipofectamine 3000 (Thermo Fisher Scientific) according to the manufacturer’s recommendation. Transfected cells were fixed and probed for viral protein staining using α-LGTV E and visualized by immunofluorescence confocal microscopy.

### Generation of lentiviruses for gene knock-down or overexpression

Short hairpin RNA (shRNA) for targeted gene knockdown was generated using the Block-IT RNAi Knockdown System (Thermo Fisher Scientific). Oligonucleotides targeting various regions of the genes of interest were annealed and the double stranded oligonucleotides were cloned into the pENTR-U6 entry vector and subsequently sequenced. Primer sequences will be available upon request. The entry vectors were further recombined into the pLenti-X2-hygro-DEST using the Gateway LR enzyme (Thermo Fisher Scientific). To recover infectious lentiviruses, the shRNA-containing vectors were transfected into HEK 293T cells along with the ViraPower Lentiviral Packaging Mix (Thermo Fisher Scientific). At 24 h post-transfection, cells were treated with 10 mM sodium butyrate and incubated for additional 48 h before lentiviruses were harvested from the cell supernatant and cell debris was removed by centrifugation (2,000 x *g* for 5 min). Lentivirus production was confirmed and semi-quantitated using the Lenti-X GoStix (Clontech). To make lentiviruses for overexpression, the gene of interest (LGTV NS5) was cloned into the lentiviral expression plasmid (pLVU-GFP) and the resultant expression vector was transfected into HEK 293T cells for virus packaging and generation of lentiviruses.

### Generation of cell lines

*P*. *leucopus* fibroblasts were transduced with lentiviruses expressing the gene of interest, control marker gene, shRNA for targeted gene knockdown or control shRNA. Cells were concurrently treated with polybrene at 6 μg/ml. At 24 h post-transduction, cells were re-transduced for an additional 6 h before replacing inoculum with complete culture medium. At 48 h post-transduction, cells were incubated in culture medium containing the appropriate drug selection: 10 μg/ml blasticidin (pLVU-GFP) or 400 μg/ml hygromycin B (pLENTI-X2-DEST) (Thermo Fisher Scientific). After passaging in selection for an additional 7–10 days, cells were plated for isolation in 48 well cluster dishes. Single clones were then expanded into individual culture flasks and tested for the gene of interest/knockdown phenotype by RT-qPCR and western blotting.

### Statistical analysis

Data were analyzed by an unpaired t test or Mann-Whitney U test using GraphPad Prism 6 software.
